# Dental Education

**Published:** 1875-06

**Authors:** L. G. Noel


### DENTAL EDUCATION.

BY L. G. NOEL, D. D. S.

At a special meeting of the Odontographic Society, held recently in the hall of the Philadelphia Dental College, a paper of mine was read upon the above subject, in which I advocated a thorough medical education for the dental student. Allusion was made to the war that has been going on between the medical and dental journals, in which the former have denied the claim of the dental profession to the position of a specialty of medicine; and to the unbecoming, begging attitude assumed by the latter. Education in the fundamental principles of medicine, was recommended, not only as the best means of putting a stop to these invidious criticisms of our medical brethren, but as absolutely essential to the faithful discharge of the duties incumbent upon the dental practitioner.

It seems that on that occasion I unwittingly got upon the toes of the editor of the Pennsylvania Dental Journal, calling forth criticism in which he accuses me of unkindness, bigotry, inexperience and lack of judgment. Having published only a synopsis of my paper, in which he does great injustice to its true spirit and intent, he arrays me in these robes, and holds me up to the derisions of his readers. I must in justice to myself say that my allusion to the course pursued by the journals, had reference more particularly to their contributors than to their editors, but Dr. Welchens takes it all home to himself, and thus a random shot has brought down game the huntsman did not see.

While it is not my desire to be always scribbling, and to keep myself ever before the readers of the dental journals, I owe it to the cause of truth in whose behalf I have endeavored to speak, as well as to myself, to correct the wrong impressions that may be be made by Dr. Welchen's remarks. He attributes to me the following sentiment, which, though I have no copy of my paper I am sure I never uttered:

"Graduate in a medical college, and you will have all the preparation you want." Now this leaves the impression upon the reader, that I would recommend attendance upon medical schools as all that is necessary to fit the student for the practice of dentistry, whereas I labored to clear the dental colleges of the obloquy that has been cast upon them, and urged their support upon the profession.

It was, to say the least, unkind of Dr. Welchens, to attack me thus, in my absence, when there were present prominent men, who advocated substantially the same views, foemen worthy of his steel whose doctrines he dared not undertake to refute. But the crowning injustice of all, was to publish a garbled synopsis of my paper, with his criticisms, in his own journal, through which I could scarcely hope to get redress. Dr. Welchens seems to take deep offense at the word "contemptible," which I intended to use in reference to all the whiners for the recognition of the medical profession, but which he fits to the editors of the dental journals, himself in particular.

It would not be hard to show that the position he takes on the education question, is, at least absurd, if not contemptible: and that is what I now propose to do. In an editorial which appeared in the Pennsylvania Dental Journal for January, 1875, he takes the following ground: "The prime and leading element in the controversy seems to be overlooked, and it is our firm conviction that until it is reognized as the main principle and starting point, no definite conclusion can be reached by the profession upon the subject of dental education. The element to which we refer is, this inordinate desire upon the part of some of our prominent men to pin dentistry to the medical profession the tendency to make the medical profession a basis for dental education, or the dental practice. Almost every writer upon this subject speaks of the dental profession as a specialty of the medical profession, thus introducing a logical contradiction or blunder every time our art is referred to as a profession. If we are a specialty of the medical profession, then, indeed, it is right and proper that we should make that profession a basis upon which to establish all preparation for the responsibilities and practice of dentistry. If we are a specialty of the medical profession, separate colleges, literature and machinery are all wrong, for with out the sanction, aid and co-operation of the institutions of learning in the medical profession, we are running into strange channels and usurping the vantage ground of other arts, and the legitimate prerogatives of other sciences. * * * * We will say in support of the position we have here taken, that there is nothing in the details and practice of dentistry which approximates the practice of medicine so near as to render the one absolutely necessary to the successful prosecution of the other. *The one* is a system having all the machinery, the appliances, education, institutions, literature and principles of practice peculiar to itself and tending to its own legitimate end and purposes. It is old, honored and powerful with its special literature, its peculiar faculty and talent, and is rightfully proud and jealous of the valuable mass of material it has thus gathered up. It takes no cognizance of the dental art. * * * * * * *The other* has its peculiar province, is a system resting upon a separate basis, and is cognizant of a separate routine, with peculiar methods of practice, special appliances and principles in its operations, its own therapeutics and pharmacopiæ, its aesthêtics, powers and destinies which belong to itself alone."

Before we proceed to quote more from this *wise counselor*, who thus brings his matured judgment to bear so heavily upon the subject, let us see if all that he has said here is true.

Dentistry is not a specialty of medicine, it has a distinct literature, peculiar methods of practice, peculiar principles, its own therapeutics and pharmacopiæ, etc., etc. This is his position. Now I would ask Dr. Welchens, if a knowledge of anatomy, chemistry and physiology is of any use to the dentist in practice. I do not think that he would deny the utility, or even the necessity of such knowledge to the dentist. Then if he ignores all connection with medicine, and everything that belongs to her, where is he going to get such knowledge? From the special literature of the dental profession of which he speaks? Our profession has given to the world no text books upon these subjects,* and if Dr. Welchens were to undertake to write them, I think he could hardly afford to ignore the facts collated by eminent anatomists, physiologists and chemists of the medical fraternity, who have left their names inscribed in living characters of truth upon almost every page of medical literature.

I challenge the editor of the Pennsylvania Journal to produce the separate and peculiar pharmacopiæ and therapeutics of which he speaks. I know that the dental profession have given some valuable discoveries to therapeutics, but the bulk of the remedies we use were discovered, their physilogical action ascertained, and their classifications made by physicians. I love dentistry, and would not filch one laurel from her fair young brow; but she must not forget her parentage; her filial respect for her old grand mother must be exacted. She is the child, the legitimate offspring of medicine, and there is no denying her maternity when she bears the likeness of her mother in every lineament and feature.

As well might you hope for the branch of a tree to flourish

*Watt's Chemistry and Handy's Anatomy are both by medical men, though prepared for the dental student. Handy was a professor in a dental college, but never a practicing dentist. Dr. Watt, though a practitioner of dentistry, was for many years a practicing physician.

after it is severed from the trunk, as to expect the science of dentistry to advance and prosper after such a lopping off of all connection with medicine, as Dr. Welchens recommends. The thing is impossible, and therefore absurd.

I do not say, nor have I ever said, that you cannot make a dentist of a student except you send him to a medical college; but I do strongly recommend students to attend upon, and graduate in a medical school, before entering a dental college. I do this because I think the course is too short in dental schools for the acquirement of all the knowledge requisite to fit a man for the practice of dentistry The two years spent in a medical school, may be devoted to the study of the fundamental principles of medicine, anatomy, physiology, chemistry and materia medica, and in acquiring a knowledge of practical surgery, by attendance upon the surgical clinics. Then upon entering a dental school, the burden which falls heaviest upon a dental student, will have been rolled off, and he is left untrammeled to apply himself to the details of dentistry proper, which will furnish him with quite enough study and practice for two more years. I take this position believing it the one best calculated to advance the profession of dentistry, and that it is the true position for every member of the profession who loves his calling, and is jealous of its progress.

The Doctor's editoral is so full of errors and contradiction, that we have not time, nor can we ask space, in which to notice them all, but there is one or two more, to which we wish to allude. After insisting that dentistry is independent of medicine in every respect, that it "rests upon a separate basis," "has its own therapeutics and pharmacopiæ," etc., he goes on to say: 'We do not presume to intimate that these two systems are the off-spring of two different principles, but they now stand, and have for years stood, not only as two different systems, but in actual antagonism in almost every respect." This is a direct admission of facts he has just most laboriously denied, and a flat contradiction of all that precedes it. It is a weak point in the wall of verbiage which he has erected around his false position, which lets out at last the truth that he is endeavoring to imprison. Does he not know that like must beget like, all through nature? Has he ever known the eggs of a wren to hatch jays? If dentistry is the off-spring of medicine, must it not necessarily rest upon the same literature as a basis? I call upon Dr. Welchens to rise and explain by what means dentistry became purged of her original iniquity, washed, born again, as it were, mounted upon a new pedestal, a separate and distinct science? Then, too, I should like him to explain in what respect the two systems are in actual antagonism, as I am unable in my stupidity to see that point.

Again he says: "The dental profession treats the diseases, and repairs the decay of the human body which comes within its sphere or jurisdiction, in a manner peculiar to itself, and which is the result of a long experience, earnest research, scientific investigation, home talent and genius, and says the medical profession is totally ignorant of all the details of the reparative system."

Will the doctor be a little more explicit? Will he not be good enough to enter a little more into details, singling out special diseases, and show what this peculiar treatment is, as applied by this distinct profession of which he speaks? Did we not learn the circulation of the blood from Harvey? And did we not from this fact gather all the phenomena of inflammation? Is inflammation of the dental tissues not to be treated just as inflammation in any other part of the body? Would not the dental surgeon remove adventitious growths from the lips and gums, just as the general surgeon would remove them from any other part of the body?

Such has been my teaching, but if Dr. Welchens is right, then is my teaching all wrong, as well as that which is being dealt out by the dental colleges whose cause he claims to espouse. Now, one more quotation of language he used before the Odontographic Society in discussing this subject, and I am done.* "We desire, most emphatically, to recognize dentistry as a profession distinct, with colleges, societies and literature peculiar to itself. It is a science of no mean

*Pennsylvania Dental Journal, May, 1875, page 191.

parts or pretensions, and of sufficient power, through this machinery, to make dental scholars of ample capacity and ability to meet all the exigencies which may present themselves. We are for drawing a strict and emphatic dividing line between dentistry and medicine, not even allowing as Prof. McQuillen does, the privilege of any to think otherwise." Now, if this is not bigotry, I don't know what to call it. He actualy denies other men the privilege of thinking for themselves.

"Upon what meat hath this our Cæsar fed, that he hath grown so great."

I grant him that there is, in our profession, abundant material of the right sort out of which schools may be made "of ample capacity and of ability to meet all the exigencies which may present themselves;" but in making up the faculties of these schools would he ignore the M. D.; A dental school without an M. D. in all its corps of teachers, would indeed be something new; that indeed would be making a radical change in our system of education. There are in America at this time ten dental colleges, employing seventy-three professors, forty-five of whom areM. D's.—more than one half. Many of these, it must be remembered, are not dentists, but regular practitioners of medicine.

Nearly all of the text books, used in these schools, were written by M. D's. but in most instances we find that their authors were the graduates of medical schools. Of these may be mentioned "Harris' Principles and Practice of Dental Surgery," "Bond's Dental Medicine," Handy's Anatomy," "Garretson's Oral Surgery," Watt's Chemical Essays," etc. The same is true of those who have contributed most largely to our journalistic literature.

In the last number of the Pennsylvania Dental Journal, notice is given of the next annual meeting of the Pennsylvania State Dental Society, at Cresson Springs, in July, in which Dr. Welchens is announced to read an essay upon the subject of "Dental Education." We advise all whose minds have been anxious and agitated over this prolonged problem to go and have them forever put to rest, for the Doctor proposes to bring his experience, matured judgment, and wise counsel to bear upon the subject. Go, anxious one, he will not even ask you to think for yourself, for this he claims as his own peculiar prerogative, all the burden of care he will roll from your mind, and assume himself.

Wondrous magnanimity.

